# A broader evaluation of vaccine-induced T cell immunity against tuberculosis

**DOI:** 10.3389/ftubr.2024.1435344

**Published:** 2024-07-19

**Authors:** Paul Ogongo

**Affiliations:** Division of Experimental Medicine, University of California, San Francisco, San Francisco, CA, United States

**Keywords:** vaccine-induced, T cells, responses, tuberculosis, evaluation

## Abstract

Although Bacillus Calmette-Guérin (BCG) vaccine, the only licensed vaccine against tuberculosis (TB), is the most widely used vaccine worldwide, TB is the second leading global killer from a single infectious agent responsible for over one million deaths annually. With the increasing threat of the emergence of drug-resistant TB, there is intense research toward better and more efficacious vaccines against TB. Indeed, TB vaccine research has blossomed in recent years: demonstration of sterilizing immunity against *Mycobacterium tuberculosis (Mtb)* challenge in non-human primates, the potential benefit of BCG revaccination in humans, and a phase IIb vaccine with ~50% efficacy against developing active disease. Consequently, several vaccines are set to begin phase 3 trials in 2024, and new candidates have entered phase 1 including mRNA-based TB vaccines. However, despite the enthusiasm, there are no known correlates of protection against TB, the antigens that induce protective immunity are incompletely defined, and the overreliance on Th1 cytokine production as an “absolute” measure of protection is increasingly debatable. In this perspective, I highlight the recent milestones in TB Vaccine research and the remaining challenges and propose suggestions for future considerations.

## Introduction

The gradual yet promising decline in global tuberculosis (TB) mortality over the past two decades has been negated by the healthcare system disruptions caused by the COVID-19 pandemic. Fortunately, the trajectory of TB-related deaths is trending downward to levels similar of the pre-COVID-19 era, with over 1.3 million deaths recorded in 2022 (1). While there have been significant improvements in shortening the treatment duration for TB, including multi-drug resistant TB (2–6), effective TB treatment is expensive, and the increasing prevalence of drug-resistant TB (including extensively drug-resistant TB) remains a cause for concern (1, 7). Consequently, the essential role of an effective vaccine against TB and the urgency to develop such vaccines to contribute to the global efforts to eliminate TB cannot be overstated. Since exposure to *Mtb* results in different infection outcomes, vaccine candidates for TB can have several indications—prevention of infection, prevention of disease, prevention of recurrence, or therapeutic (vaccine administered during the time of TB therapy to enhance immune responses targeting viable bacteria despite the presence of drugs). While emerging modeling studies suggest that the number of people with immunological evidence of *Mtb* exposure is likely overestimated (8, 9), people with latent TB (10–12) remain a large reservoir for the current global TB burden and it is believed that a post-exposure vaccine that prevents the development of TB disease will be essential in eliminating TB (13). Vaccination against TB has relied solely on BCG vaccine, the only licensed vaccine for over 100 years, usually administered at birth. BCG vaccine is effective in infants reducing the incidence of disseminated and severe TB, but it has limited efficacy against active disease in older children, adolescents, and adults (14–16). BCG vaccine also protects against other diseases through mechanisms that have only recently come to greater light (17–20). Mathematical modeling studies show that even partially efficacious new TB vaccines will significantly impact health (21, 22). In this perspective, I will discuss the recent milestones in TB Vaccine research and the remaining challenges and offer suggestions for future considerations.

## Recent advances in TB vaccine research

Since BCG is the “gold standard” vaccine against TB, the efficacy of new vaccine candidates in animal experimental studies is always benchmarked on the protection conferred by BCG vaccine or whether the subunit vaccines can augment the efficacy of BCG vaccine (23–29). Different categories of TB vaccines are in the development pipeline including live whole cell, inactivated whole cell or lysates, protein subunit, viral vector, and recently mRNA vaccines—extensively reviewed in Zhuang et al. (30), Zhou and Zhang (31), and Lai et al. (32). Here, I highlight how the knowledge gathered and discussed in published reviews can be used to comprehensively define correlates of protection against TB. Several vaccination approaches have been employed to improve the efficacy of candidate TB vaccines including different routes of administration (28, 33–41), prime-boost approaches with or without BCG as part of the vaccine schedule (42–45), as well as BCG revaccination in humans (46–50). Additionally, alternative formulations of vaccines in the preclinical stage have generated encouraging results (23, 24, 29, 51, 52) indicating that the efficacy of some previously studied vaccine candidate *Mtb* antigens can be improved upon. Significant reports using these strategies have demonstrated that it is possible to achieve sterilizing immunity against *Mtb* infection through vaccination in non-human primates—a model that more closely resembles TB disease in humans. Using the RhCMV/TB vaccine, Hansen et al. demonstrated that effective immune responses could intercept *Mtb* infection in its earlier stages, rendering complete vaccine-mediated immune control of highly pathogenic *Mtb* (28). In that study, rhesus cytomegalovirus vectors encoding *Mtb* antigen inserts (acute phase proteins 85A, 85B, and ESAT-6; latency proteins Rv1733, Rv3407, and Rv2626; and resuscitation proteins Rpf A, Rpf C, and Rpf D), RhCMV/TB—elicited and maintained highly effector-differentiated, circulating, and tissue-resident *Mtb*-specific CD4^+^ and CD8^+^ memory T cells that reduced pulmonary and extrapulmonary infection and disease by 68% compared with unvaccinated controls. Importantly, 14 out of 34 RhCMV/TB vaccinated animals showed no TB disease by computed tomography (CT) scans or at necropsy (unvaccinated controls and intradermal BCG vaccinated animals had higher necropsy scores); 10 of the vaccinated animals with no TB disease had negative *Mtb*-culture across all tissues sampled. Using different routes of BCG administration (intradermal, aerosol, and intravenous) in non-human primates, Darrah et al. demonstrated that a high-dose intravenous BCG vaccination achieved sterilizing immunity against *Mtb* challenge (40). Compared to unvaccinated animals, there was no difference in thoracic, lung, and lymph node colony forming units (CFU) in animals that received low-dose intradermal, high-dose intradermal, aerosol, and aerosol plus intradermal BCG vaccine. On the other hand, there was a significant reduction in thoracic, lung, and thoracic lymph node CFU in animals that received high-dose intravenous BCG vaccine. Most significantly, six out of 10 animals that were vaccinated intravenously had no detectable thoracic and lung CFU and only one out of 10 had thoracic lymph node CFU. In follow-up intravenous BCG dose-ranging experiments, the same group demonstrated graded immune responses and 50% protection (including sterilizing immunity) in macaques (53). Further analysis of protected vs. non-protected animals discovered that intravenous BCG-mediated protection correlated with *Mtb*-specific CD4 Th1/Th17 and NK cells in the airways. The intravenous BCG has also demonstrated efficacy against *Mtb* challenge in simian immunodeficiency virus -infected macaques, including sterilizing immunity in 9 out of 12 animals (41).

Other studies have demonstrated the superiority of mucosal BCG vaccination in mouse and non-human primate models compared to the standard intradermal route (54–58). Dijkman et al. showed that mucosal BCG vaccination prevented infection in vaccinated animals repeatedly challenged (for eight consecutive weeks) with a limiting dose of *Mtb* (1 CFU at each challenge time) on the same lung lobe, through a mechanism that involved polyfunctional Th17 cells, interleukin 10, and immunoglobulin A as the correlates of local protective immunity (59). This study demonstrated that establishing immunity at the site of infection using BCG is capable of limiting the establishment of infection and averting disease, especially with a low dose that closely resembles natural human infection (60–62).

After several disappointing results of clinical TB vaccine trials, two recent studies invigorated the enthusiasm and hope for more efficacious TB vaccines. The first was a phase 2b subunit vaccine, M72/AS01_E_ (consisting of two *Mtb* proteins PPE18 and PepA) that was administered as a prevention of disease in three countries Kenya, South Africa, and Zambia. The participants in this study were HIV-negative adults with evidence of prior *Mtb* exposure but with no clinical symptoms at the time of enrollment into the study and randomized to receive two doses of the vaccine or placebo 30 days apart and then followed for 3 years with the primary endpoint being microbiologically confirmed active, pulmonary TB with no evidence of HIV infection (63). The initial analysis done at approximately 2.3 years of follow-up showed the vaccine provided 54.0% protection against active pulmonary TB disease with no safety concerns (63). The final vaccine efficacy analysis at month 36 showed 49.7 % protection against active pulmonary TB disease in vaccine recipients compared to placebo, M72-specific antibodies and polyfunctional CD4^+^ T cells (IFNγ, TNF, or IL2 producing) increased after the first dose and were maintained throughout the follow-up period (64). The significance of this study was the demonstration for the first time that a subunit vaccine can protect *Mtb*-infected individuals from progression to active TB disease. The safety and immunogenicity of the M72 vaccine have also been evaluated in people living with HIV (PLHIV) in the MESA-TB study (Clinical Trial: NCT04556981) and found to be well-tolerated with no safety signals and found to be immunogenic in virally suppressed, ART-treated PLHIV (*Linda Han, Union World Conference on Lung Health, November 2023*). Buoyed by the phase 2b results, and after delays occasioned by the recent pandemic, the M72/AS01_E_ vaccine launched phase 3 clinical trials in March 2024 in South Africa with other centers nearing rollout as well.

The second clinical trial evaluated BCG revaccination aimed at preventing *Mtb* infection among high-risk adolescents (HIV uninfected and QuantiFERON TB test negative) (46). This randomized, partially blinded trial aimed to assess the protective effect of BCG revaccination as well as to evaluate a new recombinant protein vaccine candidate H4 (containing *Mtb* antigens Ag85B and Tb10.4) formulated with the adjuvant IC31. Since the vaccines were evaluated on interferon-gamma release assay negative (IGRA^neg^) participants, the primary endpoint was conversion from a negative to a positive IGRA, as an indicator of *Mtb* infection compared to placebo control, that is, a prevention of infection vaccine. Although none of the vaccine arms demonstrated significant protection against IGRA conversion compared to placebo, BCG revaccination showed 45.4% efficacy in preventing sustained IGRA conversion, which was interpreted as preventing latent TB infection. H4:IC31 had only 30.5% efficacy. Studies to validate the efficacy of BCG revaccination in a larger cohort are ongoing (Clinical Trial: NCT04152161) in South Africa. However, results of human BCG revaccination studies in different regions have been conflicting (49, 65, 66) partly because the endpoint in these studies is different: immunity against *Mtb* infection is not the same as immunity against TB disease. Differences in the force of infection (higher force of infection in South Africa than in Brazil), unmatched ages of study participants and prior *Mtb* sensitization could also account for the discrepancy in the results. The significance of BCG revaccination is self-explanatory: if BCG revaccination can improve the protective efficacy of this century-old vaccine in humans, we would have an inexpensive, readily deployable tool to help improve TB control globally (21, 48, 67).

Overall, TB vaccine research is in the ascendency: there are several vaccines in active phase 2b/3 clinical trials reviewed in Zhuang et al. (30), Zhou and Zhang (31), and Lai et al. (32), alternative vaccination strategies are beginning to identify immune signatures associated with vaccine-mediated protection (25, 53, 55, 59, 68), and new technologies like mRNA vaccines are being incorporated in the preclinical studies. However, the low number of TB vaccine candidates in preclinical development is a constant reminder that the TB research community needs to double the efforts to identify new targets that can create chinks in the armor of *Mtb*.

## Challenges for TB vaccine research

### Antigen selection

The complete sequencing of *Mtb* genome (69) and advances in computational biology (70, 71) have vastly increased our understanding of the complexity of *Mtb* as a pathogen from centuries of coevolution with humans. Many studies have been conducted to elucidate the identities of *Mtb* proteins and identify their role in *Mtb* survival. Relevant to vaccination, protein subunit vaccines elicit immune responses to specific antigens from the target pathogen, and since they are not viable, subunit vaccines are generally safe and can be given to immunocompromised individuals without the risk of infection. Since subunit TB vaccines target certain proteins from *Mtb*, the breadth of the immune response is narrower than for the whole organism vaccines, making the choice of antigenic target critical and an arduous task considering over 4,000 potential immunogenic proteins in the *Mtb* genome (69). An earlier *Mtb* vaccine antigen discovery study investigated 94 *Mtb* genes selected based on well-defined criteria and evaluated for IFNγ recall responses in previously *Mtb*-exposed healthy individuals demonstrated that distinct *Mtb* antigens varied in their ability to confer protection against Mtb challenge in mice (72). A recent longitudinal human cohort study in which *Mtb*-exposed individuals were followed for several years during which some participants developed TB disease (progressors) while others did not (controllers) identified antigenic peptides targeted by T cell receptor similarity groups associated with control or progression, demonstrating that distinct *Mtb* antigens are associated with infection outcomes (73). In this study, epitopes from *Mtb* antigens PE13 and CFP10 were associated with control while epitopes from EspA were associated with progression. Other TCR specificity groups were significantly enriched in progressors with incomplete protein level resolution. A study is underway to develop an mRNA vaccine containing these TCR Informed TB Antigen, TITAN, constructs of CFP10, PE13, Wbb11, and PPE18 (74). Considering the essential role of human CD4 T cell responses in controlling *Mtb* infection (75–78), it is unprecedented that the immunodominant *Mtb* antigens contain the most hyperconserved T cell epitopes, with rare exceptions (79, 80). We recently discovered that *Mtb* antigens showing evidence of diversifying evolutionary selection (80) induce predominantly Th17 responses in healthy people with a history of *Mtb* exposure (IGRA^+^) while the conserved immunodominant *Mtb* antigens induce Th1 responses (81). *Mtb* antigen availability can determine the quality of T cell responses (82); antigens expressed at high levels during the chronic phase of infection drive terminal T cell differentiation while antigens more abundant in the acute phase generate less differentiated T cells. Therefore, the selection of antigens to include as potential vaccine candidates remains a bottleneck in TB vaccine discovery (83).

Live attenuated vaccines for example MTBVAC and VPM1002, reviewed in Nieuwenhuizen et al. (84) and Martín et al. (85), provide an opportunity to overcome the challenge of distinct antigen selection as vaccine candidates because of the shared similarity in the sequence of organisms in the *Mtb* complex (MTBC) family. However, two significant considerations are necessary to overcome for future live attenuated vaccines, the safety of the vaccines and whether the attenuated bacteria still elicit the desired immune responses that could be protective.

### Correlates of protection

*Mtb* exposure results in a spectrum of infection outcomes. While symptomatic active TB is the ultimate clinical definition of the disease, with improvements in case finding and a battery of diagnostic approaches, it is increasingly recognized that there are more people with TB disease, but asymptomatic, than initially estimated (12). Defining the correlates of protection in humans is challenging because of the spectrum of infection outcomes. While evidence indicates that Th1-polarized immune responses can limit *Mtb* growth (86, 87), vaccination studies have shown that vaccine immunogenicity is not the same as a correlate of protection (88, 89). The identification of correlates of vaccine-induced protection has been hindered by the complexity of immune responses involved in the immunity to TB that includes cells of both innate and adaptive immune system (90–92). Th1 responses have been the main component in attempts to identify correlates of protection against TB, but emerging data support that Th1 cytokines alone, though essential, are insufficient for effective control of TB. IL17-producing T cells, less differentiated T cells, specific antibody isotypes, NK cells, and γδT cells have been shown to correlate with vaccine-induced protection (23–26, 53, 59, 68, 93, 94). Some immune markers that correlate with vaccine-induced protection are associated with TB clinical states, suggesting that some mediate control in natural infection (95–97). A summary of studies that identify vaccine-induced correlates of protection is shown in Table 1.

**Table 1:** Correlates of vaccine-induced T cell responses in experimental animal models.

**Vaccine description**	**Host**	**Route of vaccination**	**Immune signature**	**Measure of efficacy**	**References**
ESAT6 with LT-IIb adjuvant	Mouse	Mucosal	IL17-dependent formation of iBALT structures through induction of CXCL13	Lower lung bacterial burden compared to unvaccinated or sham vaccinated controls	(98)
Ag85B-hPIV2 vector	Mouse	Mucosal	iBALT formation with induction of Th17, and CD11b^+^CD11c^+^ cells		(99)
Subunit vaccines i – H83/CAF01: (MPT70, Rv3020c, Rv3019c and ESAT6) ii – H89/CAF01: (MPT70, Rv3020c, Rv3019c and Rv1198)	Mouse	Subcutaneous	FDS as a measure of T cell differentiation state, higher FDS means more terminally differentiated T cell repertoire. H83 vaccine rescued CD4 T cells from terminal differentiation conferring long-term protection compared to H89 vaccine. H83 vaccine specific cells had low expression of KLRG1 than saline control group.	Lower lung bacterial burden compared to Saline or BCG vaccinated controls	(94)
Subunit vaccines i-MPT70/CFA01 ii- ESAT6/CAF01	Mouse	Subcutaneous	Lower FDS for MPT70 vaccinated than ESAT-vaccinated or saline controls. Lower proportion of cytokine expressing KLRG1^+^ CD4^+^ T cells in MPT70 vaccinated compared to ESAT6 vaccinated mice.	Reduced lung bacterial burden compared to Saline controls	(100)
Subunit vaccine H107/CAF01: (PPE68, ESAT6^*^, EspI, EspC, EspA, MPT64, MPT70, MPT83)	Mouse	Subcutaneous, intravenous, or intradermal	Low FDS for H107 specific T cells. Induction of Th17 cells (IL17^+^; RORγT^+^ CD4^+^ T cells)	Reduced lung bacterial burden compared to BCG vaccinated and Unvaccinated controls	(26)
Subunit vaccine H107e/CAF01: (PPE68, ESAT6^*^, EspI^#^, EspC, EspA, MPT64, MPT70, MPT83)	Mouse	Subcutaneous	Boosted BCG specific long-lived Th17 (IL17^+^; RORγT^+^ CD4^+^) cells	Lower lung bacterial burden compared to BCG vaccinated and Unvaccinated controls	(25)
Subunit vaccine 5Ag/RR-CDG or 5Ag-ML-RR-cGAMP: (5Ag = Ag85B, ESAT6, Rv1733c, Rv2626c, and RpfD)	Mouse	Mucosal	Type I IFN independent mediated protection that includes induction of lung parenchyma CXCR3^+^ KLRG1^−^ T cells and Th1 (IFNγ^+^) and Th17 (IL17^+^) responses.	Lower lung bacterial burden compared to PBS controls or 5Ag construct without RR-CDG adjuvant.	(101)
Subunit vaccine 5Ag/ML-RR-cGAMP (CDN) or 5Ag/MLPA	Mouse	Mucosal	Induction of Th1, Th17 and Th1^*^ cells. IL17 and IFNγ dependent protection. Induction of expression of Tnfsf8 (CD153).	Reduced bacterial burden in the lung lobe compared with unvaccinated or MPLA adjuvanted vaccine	(24)
Subunit NE-TB vaccine (nanoemusion adjuvant with Ag85B and ESAT6)	Mouse	Mucosal	Induction of IL-17^+^ T-cell responses in the lungs and spleen	Reduced lung bacterial burden lower % inflammation per lung lobe Decreased chemokine (CXCL9 and CXCL2) induction. Improved B cell lymphoid follicle formation.	(23)
rIAV expressing Mtb Ag85B	Mouse	Mucosal	Induction of lung CD4^+^ TRM (CD69^+^CD11a^+^ CD44^*hi*^CD62L^*lo*^) independent of circulating memory T cells. Polyfunctional Th1 cytokines (IFNγ/TNF/IL2 or IFNγ/TNF) resident in the lung parenchyma.	Lower lung bacterial burden compared to unimmunized controls	(102)
Recombinant vaccine (SeV85AB)	Mouse	Mucosal	Induction of lung CD8^+^ TRM (CD103^+^CXCR3^+^KLRG1^−^). Induction of Ag85AB-specific polyfunctional CD8^+^ T cells (IL2^+^TNF^+^ and IFNγ^+^TNF^+^).	Lower lung bacterial burden. % reduction in inflammation per lung lobe Enhanced BCG responses in BCG prime-SeV85AB boost. Enhanced Ag85AB-specific cytotoxic CD8^+^ T cell responses.	(45)
Fusion protein CysVac2 ajuvanted with Advax (CysVac2/Advax)	Mouse	Mucosal	Induction of lung-resident (CD4^+^CD44^hi^CD62L^low^CD69^+^ IV^−^), antigen specific memory Th17 cells (IL17^+^RORγT^+^). Serum CysVac2-specific IgG1, IgG2 and IgA. Formation of iBALT structures in the lung.	Reduced bacterial burden in the lung and spleen	(103)
BCG	Mouse	Mucosal	Superior induction of *Mtb*-antigen specific CD4^+^IFNγ^+^ T cells in the lung and spleen. Induction of CD4^+^ TRM defined as CXCR3^+^PD-1^+^KLRG1^−^i.v^−^ or CD44^hi^ CD62L^lo^ CD103^+^ CD69^+^ T cells. Increased expression of transcripts typical of tissue residency *Itgae* (CD103), *Itgal* (VLA-1) and regulatory T cells (*Foxp3* and *Il10*). Increased frequency and durable *Mtb* antigen-specific CD4+ (IFNγ^+^, TNF^+^ or IL2^+^) T cells in the lung parenchyma and BAL. Enhanced proliferative capacity of lung parenchymal CD4^+^ T cells. Increased frequency of effector memory (CD44^hi^ CD62L^lo^ CD69^lo^) T cells in the lung.	Superior reduction of lung bacterial burden compared with subcutaneous vaccination and unvaccinated controls.	(56, 58, 104)
Viral vector vaccine (RhCMV/TB) encoding Mtb proteins Ag85A, Ag85B, ESAT-6, Rv1733, Rv3407, Rv2626, Rpf A, Rpf C and Rpf D.	Rhesus Macaques	Subcutaneous	Highly effector differentiated circulating and tissue resident memory Mtb-specific CD4^+^ and CD8^+^ memory T cells.	Superior reduction of bacterial burden and necropsy disease score compared with unvaccinated controls or intradermal BCG vaccination.	(28)
BCG	Rhesus Macaques	Mucosal	Local (lung) IL17A^+^CD4^+^ T cells. Production of PPD-specific IL10. Induction of IgA in the BAL.	Reduction of TB disease - Limited dissemination of disease - Reduced lung involvement (FDG uptake) - Reduced bacterial burden in the lungs and lung draining LN	(59)
BCG	Rhesus Macaques	Intravenous	TB-specific CD4 Th1/Th17 (TNF^+^IFNγ^+^ and TNF^+^IL17^+^) and NK cells in the airway. Innate cell transcription signature (type1 interferon and RIG-I-like receptor signaling pathways) at day 2 correlated with protective airway CD4 T cells (Th1/Th17) at week 8. IgM titers in the plasma and lungs.	Reduced lung inflammation (total FDG activity). Lower number of lung granuloma (serial PET-CT scans). Fewer lung bacterial burden in IV-vaccinated animals compared to other routes of vaccination.	(40, 41, 53, 68, 93)
Mtb mutant in SigH (MtbΔ*sigH)*	Rhesus Macaques	Mucosal	Recruitment of iBALT and lung CD4^+^ and CD8^+^ T cells expressing activation and proliferation markers. Strong central memory CD4^+^ and CD8^+^ T cell responses in the lung. Significant increase in polyfunctional Th1 cells (IFNγ^+^TNF^+^IL2^+^).	Reduction in bacterial burden in the lung and bronchial LN. Significantly diminished clinical manifestations (body temperature, body weight, serum CRP, thoracic radiograph scores). Reduced granulomatous pathology. Lower % lung involvement. Higher % survival compared to unvaccinated and BCG vaccinated controls.	(37)

* Fusion protein contains four copies of ESAT6;

# EspI without a proline-rich fraction (Δ75-294) for increased expression of H107e compared to the original construct H107. FDS, Functional Differentiation Score; LT-IIb, type II heat-labile enterotoxin; hPIV2, Human parainfluenza type 2 virus; CAF01, cationic adjuvant formulation 1; RR-CDG, R,R stereochemical configuration of cyclic diguanylate; ML-RR-cGAMP, mixed linkage-R,R stereochemical configuration cyclic guanosine monophosphate adenosine-monophosphate; CDN, cyclic dinucleotides; MPLA, monophosphoryl lipid A; NE, nanoemulsion based adjuvant; FDG, F-fluorodeoxyglucose; PET-CT, positron emission tomography–computed tomography; rIAV, recombinant influenza A viruses; SeV85AB, Sendai Virus expressing Mtb Ag85A and Ag8B; CysVac2, fusion protein of Mtb antigens Ag85B and CysD; CRP, C-reactive protein; BAL, Broncho-alveolar lavage; LN, lymph node.

Adjuvants are critical components of TB subunit vaccines (105–107), additional summary in Table 1, and are crucial in optimizing antigen presentation and modulating the vaccine-specific immune response. Vaccine formulations that favor Th1 responses, including induction of polyfunctional Th1 cytokines—IFNγ, TNF, and IL2 have had limited success (88). Excessive Th1 polarization favors the generation of terminally differentiated T cells that do not effectively migrate into the lung parenchyma during *Mtb* infection (94, 108, 109). T cells with Th1 and Th17 properties, referred to as Th1^*^ or Th1Th17, were associated with granuloma that restricted *Mtb* growth (110) or with asymptomatic *Mtb* infection (111) in non-human primates, suggesting the role of Th1^*^ cells in TB control. Further evidence from animal vaccination studies indicates that IL17 and Th17 responses in combination with Th1 responses are necessary for protective immunity to TB (25, 40, 53, 59). Studies in mice show that adjuvants that induce Th17 responses confer superior protection against *Mtb* challenge (23, 24, 100, 101, 112) and emerging evidence indicates that CAF^®^10b adjuvant can drive memory antibody, Th1 and Th17 vaccine-specific responses across species (113). Therefore, studies that identify correlates of vaccine-induced protection should consider the contribution of adjuvants in the formulation and ideally include an adjuvant-only group for comparisons in preclinical stages.

Since TB is a disease of the tissues, primarily the lung, immune responses at the site of infection would more likely identify correlates of protection when *Mtb* exposure does not result in active disease or correlates of risk when there is active disease. Indeed, intravenous BCG-induced airway Th1/Th17 and NK cells were recently shown to associate with protection in non-human primates (53). For ethical and practical reasons, our knowledge of immunity to TB in humans has relied mainly on studies of peripheral blood. Efforts to standardize and validate TB human infection studies to accelerate TB vaccine development are ongoing (114) with a recent report demonstrating that aerosol BCG-controlled human infection model was sufficiently well tolerated (115). Evidence from animal experimental models indicates that vaccination strategies that favor the establishment of lung tissue-resident memory (TRM) T cells often confer superior protection than vaccinations that do not generate TRM populations in the lung (116). The route of vaccination appears to play an important role in engendering TRM T cells with mucosal vaccinations more adept at generating lung TRM cells (56, 58, 59, 102, 104). However, intravenous BCG administration can also generate TRMs (40). Studies of resected human lung tissue showed that *Mtb*-responsive T cells are enriched in TB-diseased lung tissue compared to matched peripheral blood and express markers consistent with a TRM phenotype (117). Unlike in animal model studies where the time of infection with *Mtb* is known and controlled, human lung resection was done on chronic advanced TB disease patients, and the protective potential of lung TRM cells observed in this study may have been lost in this disease setting.

In summary, it is evident that no single measure of T cell immunity is the ideal correlate of protection against TB disease and thus a systems immunology approach will be vital in advancing knowledge in this area. In the context of subunit vaccines, the critical contribution of adjuvants in orchestrating immune cell interactions should be carefully evaluated.

### Other challenges

Drawing parallels from the global response to the COVID-19 pandemic, it was clear that with the right political will and available resources, it is possible to develop vaccines faster and rapidly deploy them to save lives. TB vaccine R&D is acutely underfunded (118) despite the devastation caused by TB, highlighting the lukewarm commitment by governments to tackle the disease. On the research front, the outcome of clinical trials takes several years to determine, and since the rate of progression is generally low in the community, many participants are required. In turn, this causes a delay in policy-making decisions while adding to the overall cost of TB vaccine R&D. There are efforts to use mathematical modeling to design studies to reduce the vaccine trial durations (119, 120). It is worth considering beforehand how new TB vaccines would be integrated with other control programs like treatment and diagnostics to minimize delays in distribution and address the concern of vaccine hesitancy.

## Measurement of vaccine efficacy

T cell cytokine profile is a constant consideration in evaluating vaccine-induced cellular immunity because of the central role of T cells in the control of *Mtb* infection (75–78, 121, 122). In this category, IFNγ production has been the most dominant cytokine due to the high susceptibility to TB in animals lacking IFNγ (86, 123, 124) and humans with deficiencies in IFNγ signaling (125, 126). However, IFNγ response alone is not sufficient for the control of TB (127). Several lines of evidence have shown the existence of IFNγ-independent mechanisms of T cell-mediated control of TB in animals (128, 129) and some contacts of active TB cases do not make IFNγ responses to *Mtb* antigen stimulation (130, 131). Therefore, the cytokine repertoire of vaccine-induced T cell immunity should consider T cell functions beyond IFNγ production guided by existing literature (81, 132–135).

Beyond the cytokines, CD4 T cells that express CD153 offer superior protection from *Mtb* infection across species (136–138), and expression of CD153 on *Mtb*-antigen-specific T cells is inversely associated with bacterial load and disease severity in humans (139). Studies in mice demonstrate that vaccines that favor the generation of less differentiated T cells offer superior protection (26, 94, 100). Studies that incorporate the measurement of exhaustion markers could shed more light on the phenotype of protective vaccine-induced T cells.

**Figure 1:**
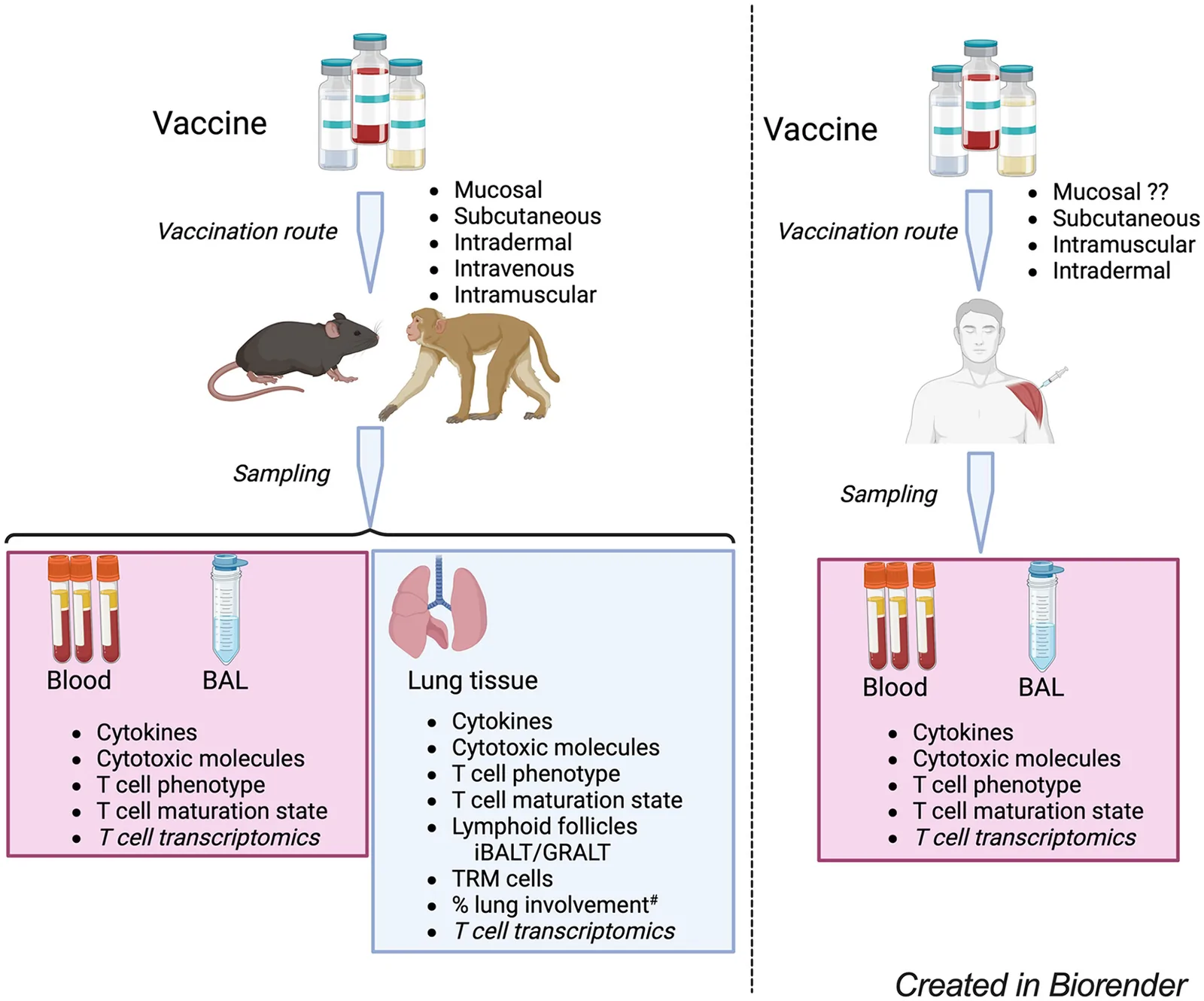
A model for the evaluation of TB vaccine-induce T cell responses in experimental models and humans. Candidate TB vaccines are delivered via various routes and samples blood, BAL – bronchoalveolar lavage – fluid and lung tissue (for experimental models) collected for analysis of vaccine-specific T cell responses. The T cell phenotype analysis considers markers of T cell lineages such as Th1, Th17, regulatory T cells as well as expression of chemokine receptors, inhibitor/exhaustion receptors. T cell maturation state analysis to consider the T cell differentiation and proliferative capacity; T cell transcriptomics to consider vaccine induced changes in the expression of T cell transcripts associated with different functions to identify novel pathways involved in vaccine response. Experimental models, analysis of % lung involvement^#^ should consider the role of T cells in tissue repair and integrity, looking at T cell expression of makers like amphiregulin that can mitigate lung tissue damage. ^#^Lung involvement can be measured by PET-CT/FCG uptake, together with microscopy approaches to define the cellular composition of the involved tissue.

Studies of *Mtb*-infected lungs provide further details of cellular organization in the lung tissue that may restrict growth and limit the dissemination of the bacteria. Lymphoid follicles [variously termed inducible bronchus-associated lymphoid tissue (iBALT) or granuloma-associated lymphoid tissue (GRALT)] are protective in mice and macaques (140–145) and have been described in resected *Mtb* infected human lung tissue (146). Vaccines can induce the formation of tertiary lymphoid structures (37, 98, 99), establish TRM populations in the lung (45, 103, 116), and provide greater *Mtb* control. It is thus evident that no single T cell feature is sufficient to define protective immunity against TB and the correlate of protection will be a T cell signature of different phenotypes and functions. Figure 1 illustrates some features to consider in defining vaccine-induced T cell responses against TB.

Evaluating vaccine efficacy is often biased toward the host responses but consideration of how vaccines restrict *Mtb* growth is also important. Enumerating CFU is the standard practice after vaccination (26, 53, 59) in animal model studies with few studies reporting the total bacterial counts as measured by bacterial chromosome equivalents (CEQ) (147–149). Similar approaches are limited in clinical settings, not least because of the number of participants involved. Intensive research on the utility of mycobacterial growth inhibition assay (MGIA)—to measure vaccine efficacy is an area of active research [extensively reviewed in Painter et al. (150)]. Finally, it is worth considering the role of vaccine induced T cell responses in mitigating lung tissue repair and integrity (151, 152) since that will have significant impact on TB transmission in humans. A host and bacteria-pronged approach to identify vaccine-induced control of *Mtb* infection will advance efforts toward defining correlates of protection against TB.

## Conclusion

The results of the Phase 2b trial of the subunit vaccine, M72/AS01E, and the number of Phase 3 clinical trials show that a new TB vaccine, with cautious optimism, is within reach. However, with very few candidates in Phase 1 and 2a stages of development, the TB vaccine research community cannot afford to take their eyes off the ball. It is important to maximize and effectively use available specimens from both experimental and clinical trials for a multifactorial approach using advances in systems immunology to improve chances of identifying correlates of vaccine-induced protection. These efforts will require the integration of innate, cellular, and humoral arms of the immune system, and resource mobilization through consortia dedicated to this cause, like the Gates Foundation-led efforts to identify correlates of M72 vaccine and BCG-revaccination studies. Human TB studies of people living with HIV, anti-TNF treatment and anti-PD1 therapy for cancer treatment have shown that TB is an immunological disease and a deeper understanding of immunity to *Mtb* infection is extremely important to evaluate and characterize new immunological correlate of protection in individuals with different immune backgrounds. To this end, new TB subunit vaccines in the clinical development stage should consider vulnerable populations, people living with HIV, people with diabetes and individuals taking biologic drugs to treat inflammatory diseases such as rheumatoid arthritis and psoriasis in the early stages for evaluation of the candidates across the heterogeneity of the population and minimize the logistical and financial costs of testing the vaccines in these populations at later time.

## Data availability statement

All relevant data is contained within the article: The original contributions discussed in the perspective are included in the article/supplementary material cited, further inquiries can be directed to the corresponding author/s.

## Ethics statement

Ethical approval was not required for the studies involving humans because the manuscript does not include original data. The studies were conducted in accordance with the local legislation and institutional requirements. The participants provided their written informed consent to participate in this study. Ethical approval was not required for the study involving animals in accordance with the local legislation and institutional requirements because there is no original data presented in this manuscript.

## Author contributions

PO: Conceptualization, Funding acquisition, Writing – original draft, Writing – review & editing, Data curation.

## References

[1] Global tuberculosis report (2023). Available online at: https://www.who.int/publications-detail-redirect/9789240083851 (accessed July 4, 2024).

[2] Sotgiu G Centis R D'ambrosio L Migliori GB. Tuberculosis treatment and drug regimens. Cold Spring Harb Perspect Med. (2015) 5:a017822. 10.1101/cshperspect.a017822 25573773 PMC4448591

[3] Kadota JL Musinguzi A Nabunje J Welishe F Ssemata JL Bishop O. Protocol for the 3HP options trial: a hybrid type 3 implementation-effectiveness randomized trial of delivery strategies for short-course tuberculosis preventive therapy among people living with HIV in Uganda. Implement Sci. (2020) 15:65. 10.1186/s13012-020-01025-8 32787925 PMC7425004

[4] Conradie F Diacon AH Ngubane N Howell P Everitt D Crook AM. Treatment of highly drug-resistant pulmonary tuberculosis. N Engl J Med. (2020) 382:893–902. 10.1056/NEJMoa1901814 32130813 PMC6955640

[5] Dorman SE Nahid P Kurbatova EV Phillips PPJ Bryant K Dooley KE. Four-month rifapentine regimens with or without moxifloxacin for tuberculosis. N Engl J Med. (2021) 384:1705–18. 10.1056/NEJMoa2033400 33951360 PMC8282329

[6] Dartois VA Rubin EJ. Anti-tuberculosis treatment strategies and drug development: challenges and priorities. Nat Rev Microbiol. (2022) 20:685–701. 10.1038/s41579-022-00731-y 35478222 PMC9045034

[7] Salari N Kanjoori AH Hosseinian-Far A Hasheminezhad R Mansouri K Mohammadi M. Global prevalence of drug-resistant tuberculosis: a systematic review and meta-analysis. Infect Dis Pover. (2023) 12:57. 10.1186/s40249-023-01107-x 37231463 PMC10210422

[8] Emery JC Richards AS Dale KD McQuaid CF White RG Denholm JT. Self-clearance of *Mycobacterium tuberculosis* infection: implications for lifetime risk and population at-risk of tuberculosis disease. Proc R Soc B. (2021) 288:20201635. 10.1098/rspb.2020.1635 33467995 PMC7893269

[9] Behr MA Edelstein PH Ramakrishnan L. Rethinking the burden of latent tuberculosis to reprioritize research. Nat Microbiol. (2024) 9:1157–8. 10.1038/s41564-024-01683-0 38671272

[10] Cohen A Mathiasen VD Schön T Wejse C. The global prevalence of latent tuberculosis: a systematic review and meta-analysis. Eur Respir J. (2019) 54:1900655. 10.1183/13993003.00655-2019 31221810

[11] Houben RMGJ Dodd PJ. The global burden of latent tuberculosis infection: a re-estimation using mathematical modelling. PLoS Med. (2016) 13:e1002152. 10.1371/journal.pmed.1002152 27780211 PMC5079585

[12] Coussens AK Zaidi SMA Allwood BW Dewan PK Gray G Kohli M. Classification of early tuberculosis states to guide research for improved care and prevention: an international Delphi consensus exercise. Lancet Respir Med. (2024) 12:484–498. 10.1016/S2213-2600(24)00028-6 38527485 PMC7616323

[13] The end TB strategy. Available online at: https://www.who.int/publications-detail-redirect/WHO-HTM-TB-2015.19 (accessed July 4, 2024).

[14] Colditz GA Brewer TF Berkey CS Wilson ME Burdick E Fineberg HV. Efficacy of BCG vaccine in the prevention of tuberculosis Meta-analysis of the published literature. JAMA. (1994) 271:698–702. 10.1001/jama.271.9.698 8309034

[15] Fine PE. Variation in protection by BCG: implications of and for heterologous immunity. Lancet. (1995) 346:1339–45. 10.1016/S0140-6736(95)92348-9 7475776

[16] Trunz BB Fine P Dye C. Effect of BCG vaccination on childhood tuberculous meningitis and miliary tuberculosis worldwide: a meta-analysis and assessment of cost-effectiveness. Lancet. (2006) 367:1173–80. 10.1016/S0140-6736(06)68507-3 16616560

[17] Singh AK Netea MG Bishai WR. BCG turns 100: its nontraditional uses against viruses, cancer, and immunologic diseases. J Clin Invest. (2021) 131:e148291. 10.1172/JCI148291 34060492 PMC8159679

[18] Cirovic B de Bree LCJ Groh L Blok BA Chan J van der Velden WJFM. BCG vaccination in humans elicits trained immunity via the hematopoietic progenitor compartment. Cell Host Microbe. (2020) 28:322–334.e5. 10.1016/j.chom.2020.05.014 32544459 PMC7295478

[19] Moorlag SJCFM Khan N Novakovic B Kaufmann E Jansen T van Crevel R. β-glucan induces protective trained immunity against *Mycobacterium tuberculosis* infection: a key role for IL-1. Cell Rep. (2020) 31:107634. 10.1016/j.celrep.2020.107634 32433977 PMC7242907

[20] Moorlag SJCFM Folkman L Horst RT Krausgruber T Barreca D Schuster LC. Multi-omics analysis of innate and adaptive responses to BCG vaccination reveals epigenetic cell states that predict trained immunity. Immunity. (2024) 57:171–187.e14. 10.1016/j.immuni.2023.12.005 38198850

[21] Clark RA Sumner T Weerasuriya CK Bakker R Scriba TJ White RG. Estimating the potential public health value of BCG revaccination. J Infect Dis. (2024) 2024:jiae089. 10.1093/infdis/jiae089 PMC11272081 39052744

[22] Sumner T Clark RA Mukandavire C Portnoy A Weerasuriya CK Bakker R. Modelling the health and economic impacts ofM72/AS01E vaccination and BCG-revaccination: Estimates for South Africa. Vaccine. (2024) 42:1311–8. 10.1016/j.vaccine.2024.01.072 38307747

[23] Ahmed M Smith DM Hamouda T Rangel-Moreno J Fattom A Khader SA. A novel nanoemulsion vaccine induces mucosal Interleukin-17 responses and confers protection upon *Mycobacterium tuberculosis* challenge in mice. Vaccine. (2017) 35:4983–9. 10.1016/j.vaccine.2017.07.073 28774560 PMC5572488

[24] Jong RM van Dis E Berry SB Nguyenla X Baltodano A Pastenkos G. Mucosal vaccination with cyclic dinucleotide adjuvants induces effective T cell homing and IL-17-dependent protection against *Mycobacterium tuberculosis* infection. J Immunol. (2022) 208:407–19. 10.4049/jimmunol.2100029 34965963 PMC8755605

[25] Dijkman K Lindenstrøm T Rosenkrands I Søe R Woodswoth JS Arlehamn CSL. A protective, single-visit TB vaccination regimen by co-administration of a subunit vaccine with BCG. NPJ Vaccines. (2023) 8:66. 10.1038/s41541-023-00666-2 37160970 PMC10169149

[26] Woodworth JS Clemmensen HS Battey H Dijkman K Lindenstrøm T Laureano RS. A *Mycobacterium tuberculosis*-specific subunit vaccine that provides synergistic immunity upon co-administration with Bacillus Calmette-Guérin. Nat Commun. (2021) 12:6658. 10.1038/s41467-021-26934-0 34795205 PMC8602668

[27] Lin PL Dietrich J Tan E Abalos RM Burgos J Bigbee C. The multistage vaccine H56 boosts the effects of BCG to protect cynomolgus macaques against active tuberculosis and reactivation of latent *Mycobacterium tuberculosis* infection. J Clin Invest. (2012) 122:303–14. 10.1172/JCI46252 22133873 PMC3248283

[28] Hansen SG Zak DE Xu G Ford JC Marshall EE Malouli D. Prevention of tuberculosis in rhesus macaques by a cytomegalovirus-based vaccine. Nat Med. (2018) 24:130–43. 10.1038/nm.4473 29334373 PMC5909823

[29] White AD Tran AC Sibley L Sarfas C Morrison AL Lawrence S. Spore-FP1 tuberculosis mucosal vaccine candidate is highly protective in guinea pigs but fails to improve on BCG-conferred protection in non-human primates. Front Immunol. (2023) 14. 10.3389/fimmu.2023.1246826 37881438 PMC10594996

[30] Zhuang L Ye Z Li L Yang L Gong W. Next-generation TB vaccines: progress, challenges, and prospects. Vaccines (Basel). (2023) 11:1304. 10.3390/vaccines11081304 37631874 PMC10457792

[31] Zhou F Zhang D. Recent advance in the development of tuberculosis vaccines in clinical trials and virus-like particle-based vaccine candidates. Front Immunol. (2023) 14. 10.3389/fimmu.2023.1238649 38022657 PMC10652786

[32] Lai R Ogunsola AF Rakib T Behar SM. Key advances in vaccine development for tuberculosis—success and challenges. NPJ Vaccines. (2023) 8:1–10. 10.1038/s41541-023-00750-7 37828070 PMC10570318

[33] Jeyanathan M Fritz DK Afkhami S Aguirre E Howie KJ Zganiacz A. Aerosol delivery, but not intramuscular injection, of adenovirus-vectored tuberculosis vaccine induces respiratory-mucosal immunity in humans. JCI Insight. (2022) 7:e155655. 10.1172/jci.insight.155655 34990408 PMC8855837

[34] Manjaly Thomas Z-R McShane H. Aerosol immunisation for TB: matching route of vaccination to route of infection. Trans R Soc Trop Med Hyg. (2015) 109:175–181. 10.1093/trstmh/tru206 25636950 PMC4321022

[35] Roediger EK Kugathasan K Zhang X Lichty BD Xing Z. Heterologous boosting of recombinant adenoviral prime immunization with a novel vesicular stomatitis virus-vectored tuberculosis vaccine. Mol Ther. (2008) 16:1161–9. 10.1038/mt.2008.59 18388911 PMC7185538

[36] Sharpe S White A Sarfas C Sibley L Gleeson F McIntyre A. Alternative BCG delivery strategies improve protection against *Mycobacterium tuberculosis* in non-human primates: Protection associated with mycobacterial antigen-specific CD4 effector memory T-cell populations. Tuberculosis (Edinb). (2016) 101:174–90. 10.1016/j.tube.2016.09.004 27865390 PMC5120991

[37] Kaushal D Foreman TW Gautam US Alvarez X Adekambi T Rangel-Moreno R. Mucosal vaccination with attenuated *Mycobacterium tuberculosis* induces strong central memory responses and protects against tuberculosis. Nat Commun. (2015) 6:8533. 10.1038/ncomms9533 26460802 PMC4608260

[38] Giri PK Sable SB Verma I Khuller GK. Comparative evaluation of intranasal and subcutaneous route of immunization for development of mucosal vaccine against experimental tuberculosis FEMS. Immunol Med Microbiol. (2005) 45:87–93. 10.1016/j.femsim.2005.02.009 15985227

[39] Wong EA Agger EM Andersen P Flynn JL. Vaccination route has an impact on level of protection of non-human primates from tuberculosis. J Immunol. (2016) 196:14621–14621. 10.4049/jimmunol.196.Supp.146.21

[40] Darrah PA Zeppa JJ Maiello P Hackney JA Wadsworth MH 2nd Hughes TK. Prevention of tuberculosis in macaques after intravenous BCG immunization. Nature. (2020) 577:95–102. 10.1038/s41586-019-1817-8 31894150 PMC7015856

[41] Larson EC Ellis-Connell AL Rodgers MA Gubernat AK Gleim JL Moriarty RV. Intravenous Bacille Calmette-Guérin vaccination protects simian immunodeficiency virus-infected macaques from tuberculosis. Nat Microbiol. (2023) 8:2080–2092. 10.1038/s41564-023-01503-x 37814073 PMC10627825

[42] Goonetilleke NP McShane H Hannan CM Anderson RJ Brookes RH Hill AVS. Enhanced immunogenicity and protective efficacy against *Mycobacterium tuberculosis* of bacille Calmette-Guérin vaccine using mucosal administration and boosting with a recombinant modified vaccinia virus. Ankara J Immunol. (2003) 171:1602–9. 10.4049/jimmunol.171.3.1602 12874255

[43] Beveridge NER Price DA Casazza JP Pathan AA Sander CR Asher TE. Immunisation with BCG and recombinant MVA85A induces long-lasting, polyfunctional *Mycobacterium tuberculosis*-specific CD4+ memory T lymphocyte populations. Eur J Immunol. (2007) 37:3089–100. 10.1002/eji.200737504 17948267 PMC2365909

[44] Lu J Wang C Zhou Z Zhang Y Cao T Shi C. Immunogenicity and protective efficacy against murine tuberculosis of a prime-boost regimen with BCG and a DNA vaccine expressing ESAT-6 and Ag85A fusion protein. Clin Dev Immunol. (2011) 2011:617892. 10.1155/2011/617892 21461375 PMC3065234

[45] Hu Z Wong K-W Zhao H-M Wen H-L Ji P Ma H. Sendai Virus Mucosal Vaccination Establishes Lung-Resident Memory CD8 T Cell Immunity and Boosts BCG-Primed Protection against TB in Mice. Mol Ther. (2017) 25:1222–33. 10.1016/j.ymthe.2017.02.018 28342639 PMC5417795

[46] Nemes E Geldenhuys H Rozot V Rutkowski K Ratangee F Bilek N. Prevention of *M. tuberculosis* Infection with H4:IC31 Vaccine or BCG Revaccination. N Engl J Med. (2018) 379:138–49. 10.1056/NEJMoa1714021 29996082 PMC5937161

[47] Rakshit S Ahmed A Adiga V Sundararaj BK Sahoo PN Kenneth J. BCG revaccination boosts adaptive polyfunctional Th1/Th17 and innate effectors in IGRA+ and IGRA- Indian adults. JCI Insight. (2019) 4:130540. 10.1172/jci.insight.130540 31743110 PMC6975271

[48] McShane H. Revaccination with BCG: does it work? Lancet Inf Dis. (2024) 24:559–560. 10.1016/S1473-3099(24)00006-9 38423022

[49] Rodrigues LC Pereira SM Cunha SS Genser B Ichihara MY de Brito SC. Effect of BCG revaccination on incidence of tuberculosis in school-aged children in Brazil: the BCG-REVAC cluster-randomised trial. Lancet. (2005) 366:1290–1295. 10.1016/S0140-6736(05)67145-0 16214599

[50] Barreto ML Pereira SM Pilger D Cruz AA Cunha SS Sant'Anna C. Evidence of an effect of BCG revaccination on incidence of tuberculosis in school-aged children in Brazil: second report of the BCG-REVAC cluster-randomised trial. Vaccine. (2011) 29:4875–4877. 10.1016/j.vaccine.2011.05.023 21616115

[51] Copland A Diogo GR Hart P Harris S Tran AC Paul MJ. Mucosal delivery of fusion proteins with bacillus subtilis spores enhances protection against tuberculosis by bacillus Calmette-Guérin. Front Immunol. (2018) 9:346. 10.3389/fimmu.2018.00346 29593708 PMC5857916

[52] Reljic R Sibley L Huang J-M Pepponi I Hoppe A Hong HA. Mucosal vaccination against tuberculosis using inert bioparticles. Infect Immun. (2013) 81:4071–80. 10.1128/IAI.00786-13 23959722 PMC3811844

[53] Darrah PA Zeppa JJ Wang C Irvine EB Buscan AN Rodgers MA. Airway T cells are a correlate of i.v. Bacille Calmette-Guerin-mediated protection against tuberculosis in rhesus macaques. Cell Host Microbe. (2023) 31:962–977. 10.1016/j.chom.2023.05.006 37267955 PMC10355173

[54] Verreck FAW Tchilian EZ Vervenne RAW Sombroek CC Kondova I Eissen OA. Variable BCG efficacy in rhesus populations: Pulmonary BCG provides protection where standard intra-dermal vaccination fails. Tuberculosis. (2017) 104:46–57. 10.1016/j.tube.2017.02.003 28454649

[55] Dijkman K Aguillo N Boot C Hofman SO Sombroek CC Vervenne RAW. Pulmonary MTBVAC vaccination induces immune signatures previously correlated with prevention of tuberculosis infection. Cell Rep Med. (2021) 2:100187. 10.1016/j.xcrm.2020.100187 33521701 PMC7817873

[56] Aguilo N Toledo AM Lopez-Roman EM Perez-Herran E Gormley E Rullas-Trincado J. Pulmonary Mycobacterium bovis BCG vaccination confers dose-dependent superior protection compared to that of subcutaneous vaccination. Clin Vaccine Immunol. (2014) 21:594–7. 10.1128/CVI.00700-13 24501340 PMC3993116

[57] Aguilo N Alvarez-Arguedas S Uranga S Marinova D Monzón M Badiola J. Pulmonary but not subcutaneous delivery of BCG vaccine confers protection to tuberculosis-susceptible mice by an interleukin 17-dependent mechanism. J Infect Dis. (2016) 213:831–9. 10.1093/infdis/jiv503 26494773

[58] Perdomo C Zedler U Kühl A Lozza L Saikali P Sander LE. Mucosal BCG vaccination induces protective lung-resident memory T cell populations against tuberculosis. MBio. (2016) 7:10-1128. 10.1128/mBio.01686-16 27879332 PMC5120139

[59] Dijkman K Sombroek CC Vervenne RAW Hofman SO Boot C Remarque EJ. Prevention of tuberculosis infection and disease by local BCG in repeatedly exposed rhesus macaques. Nat Med. (2019) 25:255–62. 10.1038/s41591-018-0319-9 30664782

[60] Plumlee CR Duffy FJ Gern BH Delahaye JL Cohen SB Stoltzfus CR. Ultra-low dose aerosol infection of mice with *Mycobacterium tuberculosis* more closely models human tuberculosis. Cell Host Microbe. (2021) 29:68–82.e5. 10.1016/j.chom.2020.10.003 33142108 PMC7854984

[61] Donald PR Diacon AH Lange C Demers A-M von Groote-Bidlingmaier F Nardell E. Droplets, dust and guinea pigs: an historical review of tuberculosis transmission research, 1878-1940. Int J Tuberc Lung Dis. (2018) 22:972–82. 10.5588/ijtld.18.0173 30092861

[62] Balasubramanian V Wiegeshaus EH Taylor BT Smith DW. Pathogenesis of tuberculosis: pathway to apical localization. Tuber Lung Dis. (1994) 75:168–78. 10.1016/0962-8479(94)90002-7 7919306

[63] Van Der Meeren O Hatherill M Nduba V Wilkinson RJ Muyoyeta M van Brakel E. Phase 2b Controlled Trial of M72/AS01E vaccine to prevent tuberculosis. N Engl J Med. (2018) 379:1621–34. 10.1056/NEJMoa1803484 30280651 PMC6151253

[64] Tait DR Hatherill M van der Meeren O Ginsberg AM van Brakel E Salaun B. Final analysis of a trial of M72/AS01E vaccine to prevent tuberculosis. N Engl J Med. (2019) 381:2429–39. 10.1056/NEJMoa1909953 31661198

[65] Dos Santos PCP Messina NL de Oliveira RD da Silva PV Puga MAM Dalcomo M. Effect of BCG vaccination against *Mycobacterium tuberculosis* infection in adult Brazilian health-care workers: a nested clinical trial. Lancet Infect Dis. (2024) 24:594–601. 10.1016/S1473-3099(23)00818-6 38423021 PMC11111441

[66] Karonga Prevention Trial Group. Randomised controlled trial of single BCG, repeated BCG, or combined BCG and killed Mycobacterium leprae vaccine for prevention of leprosy and tuberculosis in Malawi. Lancet. (1996) 348:17–24. 10.1016/S0140-6736(96)02166-6 8691924

[67] White RG Fiore-Gartland AJ Hanekom WA Vekemans J Garcia-Basteiro AL Churchyard G. What is next for BCG revaccination to prevent tuberculosis? Lancet Respir Med. (2024) 12:e7–8. 10.1016/S2213-2600(24)00009-2 38272048

[68] Irvine EB O'Neil A Darrah PA Shin S Choudhary A Li W. Robust IgM responses following intravenous vaccination with Bacille Calmette-Guérin associate with prevention of *Mycobacterium tuberculosis* infection in macaques. Nat Immunol. (2021) 22:1515–23. 10.1038/s41590-021-01066-1 34811542 PMC8642241

[69] Cole ST Brosch R Parkhill J Garnier T Churcher C Harris D. Deciphering the biology of *Mycobacterium tuberculosis* from the complete genome sequence. Nature. (1998) 393:537–44. 10.1038/31159 9634230

[70] Heupink TH Verboven L Sharma A Rennie V Fuertes MdD Warren RM. The MAGMA pipeline for comprehensive genomic analyses of clinical *Mycobacterium tuberculosis* samples. PLOS Comput Biol. (2023) 19:e1011648. 10.1371/journal.pcbi.1011648 38019772 PMC10686480

[71] Saikat ASM. Computational approaches for molecular characterization and structure-based functional elucidation of a hypothetical protein from *Mycobacterium tuberculosis*. Genomics Inform. (2023) 21:e25. 10.5808/gi.23001 37415455 PMC10326535

[72] Bertholet S Ireton GC Kahn M Guderian J Mohamath R Stride N. Identification of human T cell antigens for the development of vaccines against *Mycobacterium tuberculosis*. J Immunol. (2008) 181:7948–57. 10.4049/jimmunol.181.11.7948 19017986 PMC2586986

[73] Musvosvi M Huang H Wang C Xia Q Rozot V Krishnan A. T cell receptor repertoires associated with control and disease progression following *Mycobacterium tuberculosis* infection. Nat Med. (2023) 29:258–69. 10.1038/s41591-022-02110-9 36604540 PMC9873565

[74] Looney MM Hatherill M Musvosvi M Flynn J Kagina BM Frick M. Conference report: WHO meeting report on mRNA-based tuberculosis vaccine development. Vaccine. (2023) 41:7060–66. 10.1016/j.vaccine.2023.10.026 37872013

[75] Mogues T Goodrich ME Ryan L LaCourse R North RJ. The relative importance of T cell subsets in immunity and immunopathology of airborne *Mycobacterium tuberculosis* infection in mice. J Exp Med. (2001) 193:271–80. 10.1084/jem.193.3.271 11157048 PMC2195922

[76] Lin PL Rutledge T Green AM Bigbee M Fuhrman C Klein E. CD4 T cell depletion exacerbates acute *Mycobacterium tuberculosis* while reactivation of latent infection is dependent on severity of tissue depletion in cynomolgus macaques AIDS. Res Hum Retroviruses. (2012) 28:1693–702. 10.1089/aid.2012.0028 22480184 PMC3505050

[77] Sonnenberg P Glynn JR Fielding K Murray J Godfrey-Faussett P Shearer S. How soon after infection with HIV does the risk of tuberculosis start to increase? A retrospective cohort study in South African gold miners. J Infect Dis. (2005) 191:150–8. 10.1086/426827 15609223

[78] Kwan CK Ernst JD. HIV tuberculosis: a deadly human syndemic. Clin Microbiol Rev. (2011) 24:351–76. 10.1128/CMR.00042-10 21482729 PMC3122491

[79] Comas I Chakravartti J Small PM Galagan J Niemann S Kremer K. Human T cell epitopes of *Mycobacterium tuberculosis* are evolutionarily hyperconserved. Nat Genet. (2010) 42:498–503. 10.1038/ng.590 20495566 PMC2883744

[80] Coscolla M Copin R Sutherland J Gehre F de Jong B Owolabbi O. *M. tuberculosis* T cell epitope analysis reveals paucity of antigenic variation and identifies rare variable TB antigens. Cell Host Microbe. (2015) 18:538–48. 10.1016/j.chom.2015.10.008 26607161 PMC4758912

[81] Ogongo P Wassie L Tran A Columbus D Sharling L Ouma G. Rare Variable *M. tuberculosis*. Antigens induce predominant Th17 responses in human infection. bioRxiv 2024.03.05.583634. (2024) 10.1101/2024.03.05.583634 41591832 PMC13043089

[82] Moguche AO Musvosvi M Penn-Nicholson A Plumlee CR Mearns H Geldenhuys H. Antigen availability shapes T cell differentiation and function during tuberculosis. Cell Host Microbe. (2017) 21:695–706.e5. 10.1016/j.chom.2017.05.012 28618268 PMC5533182

[83] Young C Mkhonza MN Ogongo P. Recognition and control of *Mycobacterium tuberculosis*-infected cells: from basics to the clinic: a NIAID/WGNV workshop report 2023. Front Tubercul. (2023) 1:1303505. 10.3389/ftubr.2023.1303505 PMC13494930 42638636

[84] Nieuwenhuizen NE Kulkarni PS Shaligram U Cotton MF Rentsch CA Eisele B. The recombinant bacille calmette-guérin vaccine VPM1002: ready for clinical efficacy testing. Front Immunol. (2017) 8:1147. 10.3389/fimmu.2017.01147 28974949 PMC5610719

[85] Martín C Marinova D Aguiló N Gonzalo-Asensio J MTBVAC. a live TB vaccine poised to initiate efficacy trials 100 years after BCG. Vaccine. (2021) 39:7277–85. 10.1016/j.vaccine.2021.06.049 34238608

[86] Flynn JL Chan J Triebold KJ Dalton DK Stewart TA Bloom BR. An essential role for interferon gamma in resistance to *Mycobacterium tuberculosis* infection. J Exp Med. (1993) 178:2249–54. 10.1084/jem.178.6.2249 7504064 PMC2191274

[87] Zhang Z Fan W Yang G Xu Z Wang J Cheng Q. Risk of tuberculosis in patients treated with TNF-α antagonists: a systematic review and meta-analysis of randomised controlled trials. BMJ Open. (2017) 7:e012567. 10.1136/bmjopen-2016-012567 28336735 PMC5372052

[88] Tameris MD Hatherill M Landry BS Scriba TJ Snowden MA Lockhart S. Safety and efficacy of MVA85A, a new tuberculosis vaccine, in infants previously vaccinated with BCG: a randomised, placebo-controlled phase 2b trial. Lancet. (2013) 381:1021–8. 10.1016/S0140-6736(13)60177-4 23391465 PMC5424647

[89] Tameris M Geldenhuys H Luabeya AK Smit E Hughes JE Vermaak S. The candidate TB vaccine, MVA85A, induces highly durable Th1 responses. PLoS ONE. (2014) 9:e87340. 10.1371/journal.pone.0087340 24498312 PMC3911992

[90] Cohen SB Gern BH Urdahl KB. The tuberculous granuloma and preexisting immunity. Annu Rev Immunol. (2022) 40:589–614. 10.1146/annurev-immunol-093019-125148 35130029

[91] Nunes-Alves C Booty MG Carpenter SM Jayaraman P Rothchild AC Behar SM. In search of a new paradigm for protective immunity to TB. Nat Rev Microbiol. (2014) 12:289–99. 10.1038/nrmicro3230 24590243 PMC4085047

[92] Cooper AM. Cell-mediated immune responses in tuberculosis. Annu Rev Immunol. (2009) 27:393–422. 10.1146/annurev.immunol.021908.132703 19302046 PMC4298253

[93] Liu YE Darrah PA Zeppa JJ Kamath M Laboune F Douek DC. Blood transcriptional correlates of BCG-induced protection against tuberculosis in rhesus macaques. Cell Rep Med. (2023) 4:101096. 10.1016/j.xcrm.2023.101096 37390827 PMC10394165

[94] Clemmensen HS Knudsen NPH Billeskov R Rosenkrands I Jungersen G Aagaard C. Rescuing ESAT-6 specific CD4 T cells from terminal differentiation is critical for long-term control of murine Mtb infection. Front Immunol. (2020) 11:585359. 10.3389/fimmu.2020.585359 33240275 PMC7677256

[95] Scriba TJ Penn-Nicholson A Shankar S Hraha T Thompson EG Sterling D. Sequential inflammatory processes define human progression from *M. tuberculosis* infection to tuberculosis disease. PLoS Pathog. (2017) 13:e1006687. 10.1371/journal.ppat.1006687 29145483 PMC5689825

[96] Nathan A Beynor JI Baglaenko Y Suliman S Ishigaki K Asgari S. Multimodally profiling memory T cells from a tuberculosis cohort identifies cell state associations with demographics, environment and disease. Nat Immunol. (2021) 22:781–93. 10.1038/s41590-021-00933-1 34031617 PMC8162307

[97] Chowdhury RR Vallania F Yang Q Angel CJL Darboe F Penn-Nicholson A. A multi-cohort study of the immune factors associated with *M. tuberculosis* infection outcomes. Nature. (2018) 560:644–8. 10.1038/s41586-018-0439-x 30135583 PMC6414221

[98] Gopal R Rangel-Moreno J Slight S Lin Y Nawar HF Junecko BAF. Interleukin-17-dependent CXCL13 mediates mucosal vaccine-induced immunity against tuberculosis. Mucosal Immunol. (2013) 6:972–84. 10.1038/mi.2012.135 23299616 PMC3732523

[99] Nagatake T Wada Y Matsumoto N Shimojou M Soichiro H Nasu A. Inducible bronchus-associated lymphoid tissue plays an important role in the induction of antigen-specific immune response by Ag85B-hPIV2-based anti-tuberculosis vaccine in mice. J Immunol. (2016) 196:689. 10.4049/jimmunol.196.Supp.68.9

[100] Clemmensen HS Dube J-Y McIntosh F Rosenkrands I Jungersen G Aagaard C. In Vivo Antigen Expression Regulates CD4 T Cell Differentiation and Vaccine Efficacy against *Mycobacterium tuberculosis* Infection. MBio. (2021) 12:e00226–21. 10.1128/mBio.00226-21 33879592 PMC8092222

[101] Van Dis E Sogi KM Rae CS Sivick KE Surh NH Leong ML. STING-Activating Adjuvants Elicit a Th17 Immune Response and Protect against *Mycobacterium tuberculosis* Infection. Cell Rep. (2018) 23:1435–47. 10.1016/j.celrep.2018.04.003 29719256 PMC6003617

[102] Flórido M Muflihah H Lin LCW Xia Y Sierro F Palendira M. Pulmonary immunization with a recombinant influenza A virus vaccine induces lung-resident CD4+ memory T cells that are associated with protection against tuberculosis. Mucosal Immunol. (2018) 11:1743–52. 10.1038/s41385-018-0065-9 30115996

[103] Counoupas C Ferrell KC Ashhurst A Bhattacharyya N Nagalingam G Stewart EL. Mucosal delivery of a multistage subunit vaccine promotes development of lung-resident memory T cells and affords interleukin-17-dependent protection against pulmonary tuberculosis. NPJ Vaccines. (2020) 5:105. 10.1038/s41541-020-00255-7 33298977 PMC7665186

[104] Bull NC Stylianou E Kaveh DA Pinpathomrat N Pasricha J Harrington-Kandt R. Enhanced protection conferred by mucosal BCG vaccination associates with presence of antigen-specific lung tissue-resident PD-1+ KLRG1- CD4+ T cells. Mucosal Immunol. (2018) 12:555–64. 10.1038/s41385-018-0109-1 30446726 PMC7051908

[105] Gyu-Choi H Woong KK Jae-Shin S. Importance of adjuvant selection in tuberculosis vaccine development: Exploring basic mechanisms and clinical implications. Vaccine X. (2023) 15:100400. 10.1016/j.jvacx.2023.100400 37965276 PMC10641539

[106] Enriquez AB Izzo A Miller SM Stewart EL Mahon RN Frank DJ. Advancing Adjuvants for *Mycobacterium tuberculosis* Therapeutics. Front Immunol. (2021) 12:740117. 10.3389/fimmu.2021.740117 34759923 PMC8572789

[107] Wang H Wang S Fang R Li X Xing J Li Z. Enhancing TB vaccine efficacy: current progress on vaccines, adjuvants and immunization strategies. Vaccines (Basel). (2023) 12:38. 10.3390/vaccines12010038 38250851 PMC10820143

[108] Sakai S Kauffman KD Sallin MA Sharpe AH Young HA Ganusov VV. CD4 T cell-derived IFN-γ plays a minimal role in control of pulmonary *Mycobacterium tuberculosis* infection and must be actively repressed by PD-1 to prevent lethal disease. PLoS Pathog. (2016) 12:e1005667. 10.1371/journal.ppat.1005667 27244558 PMC4887085

[109] Sallin MA Sakai S Kauffman KD Young HA Zhu J Barber DL. Th1 differentiation drives the accumulation of intravascular, non-protective CD4 T cells during tuberculosis. Cell Rep. (2017) 18:3091–104. 10.1016/j.celrep.2017.03.007 28355562 PMC5399512

[110] Gideon HP Hughes TK Tzouanas CN Wadsworth MH 2nd Tu AA Gierahn TM. Multimodal profiling of lung granulomas in macaques reveals cellular correlates of tuberculosis control. Immun. (2022) 55:827–846.e10. 10.1016/j.immuni.2022.04.004 35483355 PMC9122264

[111] Shanmugasundaram U Buscan AN Ganatra SR Ibegbu C Quezada M Blair RV. Pulmonary *Mycobacterium tuberculosis* control associates with CXCR3- and CCR6-expressing antigen-specific Th1 and Th17 cell recruitment. JCI Insight. (2020) 5:137858. 10.1172/jci.insight.137858 32554933 PMC7453885

[112] Griffiths KL Ahmed M Shibali D Gopal R Horne W Connell TD. Targeting dendritic cells to accelerate T-cell activation overcomes a bottleneck in tuberculosis vaccine efficacy. Nat Commun. (2016) 7:13894. 10.1038/ncomms13894 28004802 PMC5192216

[113] Woodworth JS Contreras V Christensen D Naninck T Kahlaoui N Gallouët A-S. A novel adjuvant formulation induces robust Th1/Th17 memory and mucosal recall responses in Non-Human Primates. bioRxiv 2023.02.23.529651. (2023) 10.1101/2023.02.23.529651 36865310 PMC9980079

[114] Balasingam S Dheda K Fortune S Gordon SB Hoft D Kublin JG. Review of the current TB human infection studies for use in accelerating TB vaccine development: a meeting report. J Infect Dis. (2024) 7:jiae238. 10.1093/infdis/jiae238 38709726 PMC11326834

[115] Satti I Marshall JL Harris SA Wittenberg R Tanner R Ramon RL. Safety of a controlled human infection model of tuberculosis with aerosolised, live-attenuated Mycobacterium bovis BCG versus intradermal BCG in BCG-naive adults in the UK: a dose-escalation, randomised, controlled, phase 1 trial. Lancet Infect Dis. (2024) 2:143. 10.1016/S1473-3099(24)00143-9 38621405 PMC7618001

[116] Ogongo P Porterfield JZ Leslie A. Lung Tissue Resident Memory T-Cells in the Immune Response to *Mycobacterium tuberculosis*. Front Immunol. (2019) 10:992. 10.3389/fimmu.2019.00992 31130965 PMC6510113

[117] Ogongo P Tezera LB Ardain A Nhamoyebonde S Ramsuran D Singh A. Tissue-resident-like CD4+ T cells secreting IL-17 control *Mycobacterium tuberculosis* in the human lung. J Clin Invest. (2021) 131:142014. 10.1172/JCI142014 33848273 PMC8121523

[118] Venkatesan P. Worrying lack of funding for tuberculosis. Lancet Infect Dis. (2022) 22:318. 10.1016/S1473-3099(22)00073-1 35218748 PMC8865854

[119] Garcia-Basteiro AL White RG Tait D Schmidt AC Rangaka MX Quaife M. End-point definition and trial design to advance tuberculosis vaccine development. Eur Respir Rev. (2022) 31:220044. 10.1183/16000617.0044-2022 35675923 PMC9488660

[120] Hill PC Cobelens F Martinez L Behr MA Churchyard G Evans T. An Aspiration to Radically Shorten Phase 3 Tuberculosis Vaccine Trials. J Infect Dis. (2023) 228:1150–3. 10.1093/infdis/jiad356 37607272

[121] Scanga CA Mohan VP Yu K Joseph H Tanaka K Chan J. Depletion of CD4(+) T cells causes reactivation of murine persistent tuberculosis despite continued expression of interferon gamma and nitric oxide synthase 2. J Exp Med. (2000) 192:347–58. 10.1084/jem.192.3.347 10934223 PMC2193220

[122] Lawn SD Myer L Edwards D Bekker L-G Wood R. Short-term and long-term risk of tuberculosis associated with CD4 cell recovery during antiretroviral therapy in South Africa. AIDS. (2009) 23:1717–25. 10.1097/QAD.0b013e32832d3b6d 19461502 PMC3801095

[123] Cooper AM Dalton DK Stewart TA Griffin JP Russell DG Orme IM. Disseminated tuberculosis in interferon gamma gene-disrupted mice. J Exp Med. (1993) 178:2243–7. 10.1084/jem.178.6.2243 8245795 PMC2191280

[124] Green AM Difazio R Flynn JL. IFN-γ from CD4 T cells is essential for host survival and enhances CD8 T cell function during *Mycobacterium tuberculosis* infection. J Immunol. (2013) 190:270–7. 10.4049/jimmunol.1200061 23233724 PMC3683563

[125] van de Vosse E Haverkamp MH Ramirez-Alejo N Martinez-Gallo M Blancas-Galicia L Metin A. IL-12Rβ1 deficiency: mutation update and description of the IL12RB1 variation database. Hum Mutat. (2013) 34:1329–39. 10.1002/humu.22380 23864330 PMC4104692

[126] Filipe-Santos O Bustamante J Chapgier A Vogt G de Beaucoudrey L Feinberg J. Inborn errors of IL-12/23- and IFN-gamma-mediated immunity: molecular, cellular, and clinical features. Semin Immunol. (2006) 18:347–61. 10.1016/j.smim.2006.07.010 16997570

[127] Vilaplana C Prats C Marzo E Barril C Vegué M Diaz J. To achieve an earlier IFN-γ response is not sufficient to control *Mycobacterium tuberculosis* infection in mice. PLoS ONE. (2014) 9:e100830. 10.1371/journal.pone.0100830 24959669 PMC4069189

[128] Gallegos AM van Heijst JWJ Samstein M Su X Pmer EG Glickman MS. A gamma interferon independent mechanism of CD4 T cell mediated control of M tuberculosis infection in vivo. PLoS Pathog. (2011) 7:e1002052. 10.1371/journal.ppat.1002052 21625591 PMC3098235

[129] Van Dis E Fox DM Morrison HM Fines DM Babirye JP McCann LH. IFN-γ-independent control of *M. tuberculosis* requires CD4 T cell-derived GM-CSF and activation of HIF-1α. PLoS Pathog. (2022) 18:e1010721. 10.1371/journal.ppat.1010721 35877763 PMC9352196

[130] Lu LL Smith MT Yu KKQ Luedemann C Suscovich TJ Grace PS Cain A. IFN-γ-independent immune markers of *Mycobacterium tuberculosis* exposure. Nat Med. (2019) 25:977–87. 10.1038/s41591-019-0441-3 31110348 PMC6559862

[131] Davies LRL Smith MT Cizmeci D Fischinger S Lee JS-L Lu LL. IFN-γ independent markers of *Mycobacterium tuberculosis* exposure among male South African gold miners. eBioMedicin. (2023) 93:104678. 10.1016/j.ebiom.2023.104678 37379655 PMC10320233

[132] Rothchild AC Jayaraman P Nunes-Alves C Behar SM iNKT. cell production of GM-CSF controls *Mycobacterium tuberculosis*. PLoS Pathog. (2014) 10:e1003805. 10.1371/journal.ppat.1003805 24391492 PMC3879349

[133] Li L Qiao D Fu X Lao S Zhang X Wu C. Identification of *Mycobacterium tuberculosis*-specific Th1, Th17 and Th22 cells using the expression of CD40L in tuberculous pleurisy. PLoS ONE. (2011) 6:e20165. 10.1371/journal.pone.0020165 21625607 PMC3097245

[134] Bunjun R Omondi FMA Makatsa MS Keeton R Wendoh JM Müller TL. Th22 Cells Are a Major Contributor to the Mycobacterial CD4+ T Cell Response and Are Depleted During HIV Infection. J Immunol. (2021) 207:1239–49. 10.4049/jimmunol.1900984 34389623 PMC8387408

[135] Gopal R Monin L Slight S Uche U Blanchard E Junecko BAF. Unexpected role for IL-17 in protective immunity against hypervirulent *Mycobacterium tuberculosis* HN878 infection. PLoS Pathog. (2014) 10:e1004099. 10.1371/journal.ppat.1004099 24831696 PMC4022785

[136] Sallin MA Kauffman KD Riou C Du Bruyn E Foreman TW Sakai S. Host resistance to pulmonary *Mycobacterium tuberculosis* infection requires CD153 expression. Nat Microbiol. (2018) 3:1198–205. 10.1038/s41564-018-0231-6 30202016

[137] Foreman TW Nelson CE Sallin MA Kauffman KD Sakai S Otaizo-Carrasquero F. CD30 co-stimulation drives differentiation of protective T cells during *Mycobacterium tuberculosis* infection. J Exp Med. (2023) 220:e20222090. 10.1084/jem.20222090 37097292 PMC10130742

[138] Gress AR Bold TD. TB granuloma: CD30 co-stimulation for CD4+ T cell co-operation. J Exper Med. (2023) 220:e20230547. 10.1084/jem.20230547 37158981 PMC10174186

[139] Du Bruyn E Ruzive S Arlehamn CSL Sette A Sher A Barber DL. *Mycobacterium tuberculosis*-specific CD4 T cells expressing CD153 inversely associate with bacterial load and disease severity in human tuberculosis. Mucosal Immunol. (2021) 14:491–9. 10.1038/s41385-020-0322-6 32678272 PMC7855386

[140] Slight SR Rangel-Moreno J Gopal R Lin Y Junecko BAF Mehra S. CXCR5^+^ T helper cells mediate protective immunity against tuberculosis. J Clin Invest. (2013) 123:712–26. 10.1172/JCI65728 23281399 PMC3561804

[141] Dunlap MD Prince OA Rangel-Moreno J Thomas KA Scordo JM Torrelles JB. Formation of Lung Inducible Bronchus Associated Lymphoid Tissue Is Regulated by *Mycobacterium tuberculosis* Expressed Determinants. Front Immunol. (2020) 11:1325. 10.3389/fimmu.2020.01325 32695111 PMC7338767

[142] Marin ND Dunlap MD Kaushal D Khader SA. Friend or Foe: The Protective and Pathological Roles of Inducible Bronchus-Associated Lymphoid Tissue in Pulmonary Diseases. J Immunol. (2019) 202:2519–26. 10.4049/jimmunol.1801135 31010841 PMC6481307

[143] Swanson RV Gupta A Foreman TW Lu L Choreno-Parra JA Mbandi SK. Antigen-specific B cells direct T follicular-like helper cells into lymphoid follicles to mediate *Mycobacterium tuberculosis* control. Nat Immunol. (2023) 24:855–68. 10.1038/s41590-023-01476-3 37012543 PMC11133959

[144] Foreman TW Nelson CE Kauffman KD Lora NE Vinhaes CL Dorosky DE. CD4 T cells are rapidly depleted from tuberculosis granulomas following acute SIV co-infection. Cell Rep. (2022) 39:110896. 10.1016/j.celrep.2022.110896 35649361

[145] Kauffman KD Sallin MA Sakai S Kamenyeva O Kabat J Weiner D. Defective positioning in granulomas but not lung-homing limits CD4 T-cell interactions with *Mycobacterium tuberculosis*-infected macrophages in rhesus macaques. Mucosal Immunol. (2018) 11:462–73. 10.1038/mi.2017.60 28745326 PMC5785573

[146] Krause R Ogongo P Tezera L Ahmed M Mbano I Chambers M. B Cell Heterogeneity in Human Tuberculosis Highlights Compartment-Specific Phenotype and Putative Functional Roles. (2023). 10.21203/rs.3.rs-3337492/v1 38755239 PMC11099031

[147] Smith CM Baker RE Proulx MK Mishra BB Long JE Park SW. Host-pathogen genetic interactions underlie tuberculosis susceptibility in genetically diverse mice. Elife. (2022) 11:e74419. 10.7554/eLife.74419 35112666 PMC8846590

[148] Lin PL Ford CB Coleman MT Myers AJ Gawande R Ioerger T. Sterilization of granulomas is common in active and latent tuberculosis despite within-host variability in bacterial killing. Nat Med. (2014) 20:75–9. 10.1038/nm.3412 24336248 PMC3947310

[149] Muñoz-Elías EJ Timm J Botha T Chang W-T Gomez JE McKinney JD. Replication dynamics of *Mycobacterium tuberculosis* in chronically infected mice. Infect Immun. (2005) 73:546–51. 10.1128/IAI.73.1.546-551.2005 15618194 PMC538940

[150] Painter H Harriss E Fletcher HA McShane H Tanner R. Development and application of the direct mycobacterial growth inhibition assay: a systematic review. Front Immunol. (2024) 15:1355983. 10.3389/fimmu.2024.1355983 38380319 PMC10877019

[151] Arpaia N Green JA Motedo B Arvey A Hemmers S Yuan S. A distinct function of regulatory T cells in tissue. Protection Cell. (2015) 162:1078–89. 10.1016/j.cell.2015.08.021 26317471 PMC4603556

[152] Zaiss DMW Gause WC Osborne LC Artis D. Emerging functions of amphiregulin in orchestrating immunity, inflammation, and tissue repair. Immunity. (2015) 42:216–26. 10.1016/j.immuni.2015.01.020 25692699 PMC4792035

